# Time Sequence of Measurement Affects Blood Pressure Level in an African American Cohort

**DOI:** 10.51894/001c.30124

**Published:** 2022-02-24

**Authors:** Michael Marshall, Nancy Jackson, Brittni McClellan, Max Zlatopolsky, Susan Steigerwalt, Grace D. Brannan

**Affiliations:** 1 Department of Internal Medicine Ascension Providence Hospital; 2 Department of Biomedical Research Ascension Providence Hospital; 3 St. Vincent Medical Center-Mercy Health; 4 GDB Research and Statistical Consulting

**Keywords:** blood pressure, time sequence, African American, automated office blood pressure

## Abstract

**INTRODUCTION:**

Uncontrolled hypertension can result in severe clinical conditions such as stroke, chronic kidney disease and congestive heart failure, especially in African American populations. To the knowledge of the authors, the effect of time sequence on blood pressure (BP) using an Automated Office Blood Pressure (AOBP) device has not been documented in an African American cohort. The objective of this study was to investigate the possible influence of time sequence of measurement (pre- and post-physician visit) on BP readings in an African American cohort, in the presence or absence of a Medical Assistant (MA) via AOBP monitoring.

**METHODS:**

A two-phase, single-blinded, non-randomized trial was conducted at MI-based Ascension Providence Hospital with a convenience sample of hypertensive patients. BP readings were taken using both an Omron 907 (Omron Corp., Kyoto, Japan) and a Welch Allyn (WA) Connex Spot Monitor (Welch Allyn, Inc., Skaneateles Falls, NY) AOBP devices. Descriptive statistics were generated, and T-tests were performed.

**RESULTS:**

In Phase 1, (N = 148), the mean systolic/diastolic readings for the pre-physician visits (141/82 mmHg) were statistically significantly higher than the post-visit readings (134/80 mmHg) (p ≤ 0.02). Post-visit physician readings from either AOBP device did not differ statistically (p = 0.72). In Phase 2 (n = 50), the presence of an MA resulted in significantly higher readings than when an MA was absent, however, the results of Phase 2 also supported the trends for lower BP post-physician visit found in Phase 1.

**CONCLUSION:**

Based on the consistency of these results, a post-physician visit AOBP reading, in the presence or absence of an MA, may provide a more accurate BP measurement to determine whether or not to treat hypertension in African American patients.

## INTRODUCTION

Hypertension is defined as a systolic blood pressure (BP) greater than 130 mmHg or diastolic BP greater than 80 mmHg or taking medicine for hypertension. It is one of the most frequently encountered conditions in adult primary care, with a higher prevalence and greater disease burden in African American populations.^1^ More specifically, the overall prevalence of hypertension among African Americans is 39.1% while in non-Hispanic Whites it is 28.5%.^1^ Uncontrolled hypertension can result in severe, sometimes life-threatening conditions including stroke, chronic kidney disease, and congestive heart failure.^2^

Automated Office Blood Pressure (AOBP) monitoring is a method of obtaining blood pressure with a single or multiple reading automated system allowing patients to rest comfortably prior to measurements being obtained.^3^ This method creates a delay in the BP measurement, allowing for staff to be absent for five minutes prior to readings being taken. A more involved assessment, ambulatory BP monitoring (ABPM) assesses daytime and nighttime blood pressure during routine daily activities typically for one 24-hour period, and of all measuring techniques is considered a gold standard as it correlates best with target (i.e., heart, kidney, and brain) organ damage and mortality.^4^ AOBP readings had been demonstrated as not significantly different from those measure using ABPM.^5^

BP is traditionally measured at the beginning of an office visit, prior to seeing a physician. African American individuals have been historically underrepresented in clinical trials assessing blood pressure diagnosis/treatment, despite shouldering a greater proportion of the overall disease burden.^6^ In addition, the efficacy of AOBP measurement has not been well studied in the African American population.

Complicating BP measurement is the fact that the presence of clinic staff while measurement is taken may itself increase BP. Sometimes referred to as “Whitecoat Hypertension,” this can be a common phenomenon associated with overestimation of a patient’s true average BP, leading to difficulties in appropriately diagnosing and treating patient hypertension.^7^ Accurate measurement of BP in the clinic is critical for maintaining good control and preventing the over or under-treatment of hypertension.^8^ The authors were unable to identify previous literature specifically examining the effect of time sequence on BP measurements for an African American cohort, such as comparing BP measurements before and after the physician encounter, as well as with the presence or absence of a medical assistant (MA).

## Purpose of Study

The purpose of this study was to determine the effect of time sequence in the measurement (pre- and post-physician visit) of blood pressure in an African American cohort in the presence or absence of a MA. More specifically, the objectives of this study were twofold: 1) to determine the concordance of single vs multiple reading automated BP devices pre- and post-physician visit without a clinic staff (i.e., MA) present; and 2. to determine whether the presence of a MA altered the concordance of the AOBP measurements pre- and post-physician visit.

## METHODS

### Study Design

Prior to data collection, the study was approved by Ascension Providence IRB. From April 2017 to December 2018 a two-phase, prospective, single-blinded and non-randomized trial was conducted at the Ascension Providence Hospital Academic Internal Medicine clinic.

### Sample Population

A convenience sample of male and female African American patients who were 18 years and older with a previous diagnosis of hypertension who were attending clinic for a routine visit were recruited and consented. Exclusion criteria included history of end-stage renal disease with an arterio-venous fistula (i.e., an abnormal connection between an artery and a vein), arm circumference >20 cm or < 7 cm, history of breast cancer with previous mastectomy or upper extremity lymphedema, as the presence of any of these factors could have skewed sample BP readings. All eligible patients coming for their scheduled visits were screened by an MA and asked if they wanted to participate in the study. Once a potential participant was identified, the MA provided a study information sheet. If the patient agreed to participate, a written consent was obtained in the exam room.

### Study Procedure

#### Phase 1

During the Phase 1 pre-physician visit period (intake), patients were placed in an exam room, feet on the floor, and an initial single measurement BP (“Pre Omron single)” was taken by an MA using an Omron 907 (Omron Corp., Kyoto, Japan) AOBP device. Immediately following their physician visit, two measures were done. First, a series of three unobserved measurements were obtained within a five-minute pre-programmed interval using a Welch Allyn (WA) Connex Spot Monitor (Welch Allyn, Inc., Skaneateles Falls, NY) device while the patient was alone in the room. The three readings were averaged (Post WA multiple). Second, this was followed by a final single measurement BP (“Post Omron single”) by the MA. The two post readings were used to determine concurrence between the two devices.

#### Phase 2

All BP measurements during Phase 2 were conducted in the same manner as Phase 1, but on two groups of patients. To determine the possibility of Whitecoat effect, one group had an MA in the room (i.e., “MA in”) during the WA device measurements. The second group had the “MA out of the room (i.e.,”MA out)” during the automated WA BP measurements. Patients in the MA out group were under the care of one physician and patients in the MA in group were under the care of another physician.

### Additional Study Measures

In addition to the blood pressure readings, demographic & medical history were abstracted from patients’ charts. This included: age, gender, height, weight, BMI, major comorbidities (e.g., obesity, Diabetes Mellitus (DM), cardiovascular disease, smoking, obstructive sleep apnea, and whether or not the patient was receiving prescribed blood pressure medications.

### Data Analyses

Descriptive statistics were generated, and inferential statistical analyses were conducted by the second author (NJ) using *SPSS Version 23* (IBM SPSS Statistics for Windows. Armonk, NY: IBM Corp). Statistical significance was set at p < 0.05. During Phase 1 and Phase 2, a two-tailed paired Student’s t-test was performed to compare differences in sample patients’ blood pressure readings within a subgroup. During Phase 2, an independent Student’s t test was performed to compare differences between patient subgroups.

## RESULTS

### Phase 1

A total of N = 148 African American patients with a mean age of 59.5 (SD = 13.2 years) were included, 88 (59.5%) of which were female. Patients’ average BMI was 33.4 (SD = 13.5). The sample has documented diabetes as the most common comorbidity, with 47 (32%) patients having this condition. A total of 33 (22%) of patients were smokers, while 10 (7%) had obstructive sleep apnea. As expected, most patients (i.e., 119 (80%)) were on at least one prescribed BP medication, while 76 (51%) were on calcium channel blocker (CCB) and 69 (47%) were on diuretics as additional medications (Table 1).

**Table 1.** Phase 1: Demographic, comorbidities, and blood pressure medication information of 148 patients. Data are expressed in frequency, percentages, or mean ± standard deviation (SD).

**Factor**	**Measure**
Demographic	
Sex (male)	60 (40.5%)
Age (years)	59.5 (SD = 13.2)
Height (meters)	1.70 (SD = 0.13)
Weight (kilograms)	93.8 (SD = 24.0)
BMI	33.4 (SD = 13.5)
Comorbidities	
Diabetes Mellitus	47 (32%)
Coronary Artery Disease (CAD)	19 (13%)
Smoker	32 (22%)
Obstructive Sleep Apnea	10 (7%)
Blood Pressure Medicines	
Calcium channel blocker (CCB)	76 (51%)
Diuretic	69 (47%)
Angiotensin-converting enzyme (ACE)	40 (27%)
Angiotensin receptor blocker (ARB)	29 (20%)
Any BP medication	119 (80%)

Table 2 depicts systolic and diastolic blood pressure reading patterns. The mean systolic BP (Pre Omron single) was statistically significantly higher by over seven points (140.87 mmHg) compared to the Post WA multiple (133.51 mmHg, p < 0.01) and Post Omron single (133.41 mmHg, p < 0.01). The Post WA multiple and Post Omron single systolic BP were not statistically significantly different (p = 0.92).

The same trends were observed with sample diastolic readings, where the Pre Omron single was higher by at least 1.67 points compared to the Post WA multiple (p = 0.02) and Post Omron single (p = 0.02). The Post WA multiple (80.22 mmHg) and Post Omron single (80.02 mmHg) diastolic readings were not statistically significantly different (p = 0.73).

**Table 2.** Phase 1 systolic and diastolic blood pressure readings.

**Blood Pressure Measure**	**Sequence and Device**			**Is the Pre Omron single different from the Post Omron single?** **(p value)**	**Is the Post WA multiple different from the Pre Omron single?** **(p value)**	**Is the Post Omron single different from the Post WA multiple** **(p value)?**
	**Pre Omron single**	**Post WA multiple**	**Post Omron single**			
**Systolic Mean (mmHg)**	140.87	133.51	133.41	**< 0.01**	**< 0.01**	0.92
**Systolic Standard Deviation (mmHg)**	22.85	19.03	21.03			

**Diastolic Mean (mmHg)**	81.89	80.22	80.02	**0.02**	**0.02**	0.73
**Diastolic Standard Deviation (mmHg)**	13.17	10.09	11.27			

### Phase 2

When the two groups (i.e., MA in and MA out) were compared, there was no statistically significant difference by gender (p = 0.25), BMI (p = 0.16), or by comorbidities of diabetes mellitus, coronary artery disease, smoking or hypertension (Table 3). In addition, there was no significant difference across types of hypertension medications taken. However, there was a significant difference in mean age of subjects for MA in the room, 56 (SD = 10), who were younger, compared to mean MA out of the room, 67 (SD = 10), p < 0.01 (Table 3).

**Table 3.** Phase 2: Demographic, comorbidities and blood pressure medication information of patients (N = 25 each group). Data are expressed in frequency, percentages, or mean ± standard deviation.

**Factors**	**MA In Group**	**MA Out Group**	**p-value for MA In vs MA Out**
**Demographics**			
**Sex (male)**	13 (52%)	8 (32%)	0.25
**Age (years)**	56 (SD = 10)	67 (SD = 10)	**< 0.01**
**Height (meters)**	1.70 (SD = 0.10)	1.65 (SD = 0.08)	**0.02**
**Weight (kilograms)**	88.9 (SD = 22.7)	90.3 (SD = 23.6)	0.83
**BMI**	30.5 (SD = 6.5)	33.0 (SD = 8.3)	0.16
**Comorbidities**			
**Diabetes Mellitus**	8 (32%)	9 (36%)	1.0
**Coronary Artery Disease (CAD)**	1 (4%)	2 (8%)	1.0
**Smoker**	4 (16%)	1 (4%)	0.35
**Obstructive Sleep Apnea**	2 (8%)	0	0.50
**Blood Pressure Medicines**			
**Calcium channel blocker (CCB)**	15 (60%)	17 (68%)	0.80
**Diuretic**	7 (28%)	7 (28%)	1.0
**Angiotensin-converting enzyme (ACE)**	6 (24%)	4 (16%)	0.73
**Angiotensin receptor blocker (ARB)**	6 (24%)	7 (28%)	1
**Any BP medication**	19 (76%)	24 (96%)	0.10

When comparing the two subgroups, a significant difference in all BP readings were noted (Table 4). For all measurements taken, the MA in group had higher average blood pressures compared to the MA out group. Despite this, the Pre Omron single reading was always higher than readings taken at the end of the patient visit (Post WA multiple and Post Omron single).

**Table 4.** Phase 2: Mean blood pressure readings of patients with the Medical Assistant in (MA In) and out (MA Out) of the room.

**Sequence and Device**	BP Measurement	**MA In Group**	**MA Out Group**	**MA In vs MA Out**
		**Mean (mmHg)**	**Mean (mmHg)**	**p-value**
**Pre Omron single**	Systolic	144.7 (SD = 22.0)	132.6 (SD = 15.7)	**0.03**
	Diastolic	86.8 (SD = 11.5)	74.2 (SD = 10.6)	**< 0.01**
**Post WA multiple**	Systolic	138.53 (SD = 21.7)	124.2 (SD = 14.5)	**< 0.01**
	Diastolic	84.28 (SD = 11.6)	72.1 (SD = 7.4)	**< 0.01**
**Post Omron single**	Systolic	139.84 (SD = 23.6)	124.4 (SD = 14.2)	**0.01**
	Diastolic	84.64 (SD = 11.4)	72.3 (SD = 7.3)	**< 0.01**

*Sample size for each group is N = 25.

Table 5 shows the mean change in sample patients’ blood pressure readings for each of the two subgroups: the Medical Assistant in (MA In) and out (MA Out) of the room. In the MA in subgroup, Pre Omron single vs Post WA multiple resulted in a statistically significant difference in mean systolic readings (p < 0.01), although not in diastolic (p = 0.06) BP readings. The Pre vs Post Omron single readings were not statistically significant for both systolic (p = 0.07) and diastolic (p = 0.11) BP readings. The Post Omron single vs Post WA multiple were not statistically significant for systolic (p = 0.52) and diastolic (p = 0.50) BP readings.

**Table 5.** Phase 2: Mean difference between blood pressure readings of patients within each of the two groups: the Medical Assistant in (MA In) and out (MA Out) of the room.

**Sequence and Device**	**BP Measurement**	**MA In Group**		**MA Out Group**	
		**Difference (mmHg)**	**p value**	**Difference (mmHg)**	**p value**
**Pre Omron single vs. Post WA multiple**	**Systolic**	6.15	**< 0.01**	8.35	< **0.01**
	**Diastolic**	2.52	0.06	2.07	0.21
**Pre vs. Post Omron single**	**Systolic**	4.84	0.07	8.2	**< 0.01**
	**Diastolic**	2.16	0.11	1.88	0.25
**Post Omron single vs. Post WA multiple**	**Systolic**	-1.31	0.52	-0.15	0.32
	**Diastolic**	-0.36	0.47	-0.19	< **0.01**

In the MA out group, Pre Omron single vs Post WA multiple resulted in a statistically significant difference in systolic (p < 0.01) but not in diastolic (p = 0.21) BP readings. The Pre vs Post Omron single readings yielded a statistically significant difference in systolic (p < 0.01) but not in diastolic (p = 0.25) BP reading. The differences in Post Omron single vs Post WA multiple were not statistically significant for systolic (p = 0.32) but were statistically significant for diastolic (p < 0.01) BP readings.

## DISCUSSION

In summary, Phase 1 demonstrated a significant difference between pre- and post- physician visit BP readings, indicating that the sequence of BP measurement could be an important factor. Phase 2, confirmed Phase 1 in that all post - physician measures were lower compared to pre-physician visit BP measures. During Phase 2, patients in both groups exhibited significantly lower post-physician systolic BP readings compared to their pre-physician visit. The diastolic, although trending towards lower post BP readings, did not reach statistical significance. We have concluded that this may be due at least in part to our smaller sample size.

In addition, our initial findings indicated that the multiple readings with Post WA multiple were not statistically different when compared to the Post Omron single reading. This may be important since AOBP with multiple reading devices has been correlated with ABPM.^8^ ABPM is considered the BP monitoring gold standard for elevated BP.^9,10^ This may also indicate that the post-physician visit measurements may provide truer BP measures for determining whether to treat hypertension.

ABPM was superior for predicting clinical outcomes.^9^ Although ABPM is considered the gold standard, it may be cost prohibitive and logistically difficult in many primary care clinic settings.^9^ Using AOBP represents measurement in a real-world setting that is less expensive. In addition, AOBP has been adopted by numerous international organizations supporting it as a “better” approach to measuring BP.^8,11–13^

In terms of Whitecoat effect, we found some evidence for this in Phase 2 results in this population of patients. The Whitecoat effect is consistent with what is found in the literature.^4^ For example, this residual effect was sustained when comparing the mean difference in systolic BP reading from Pre Omron single in the MA in group (4.84 mmHg) to the Post Omron single in the MA out group (8.20 mmHg).

This information can be potentially beneficial to our clinic and others since most BP measurements are taken at the beginning of the visit. These study findings may help improve accuracy and prevent over or under treatment of hypertension. For example, post-physician AOBP monitoring without an MA present may be useful in African American predominant areas and lead to greater blood pressure measurement accuracy, diagnosis, and improved hypertension treatment.

### Study Limitations

This study has several limitations. We did not randomize the sequence of Omron single and WA multiple device readings during post- physician visit measurements. In addition, although we saw a similar trend between Phase 1 and 2 results, the small number of patients in Phase 2 yielded statistically inconclusive results. Also, age differences between the MA in and MA out subgroups (i.e., MA in patients significantly younger) may have skewed our results.

Future studies could include a trial which measures a Pre and a Post Omron single reading coupled with Pre and a Post WA reading. This would be to exclude the possibility that the phenomenon we are observing is related to multiple readings. It is also important to confirm the correlation between the Post WA multiple and Post Omron single readings at the end of the visit. A subsequent RCT to compare end of office visit readings to either home blood pressure readings or 24-hour ambulatory monitoring is also recommended.

## CONCLUSIONS

Based on these study results, measuring a patients’ blood pressure a second time at the end of their office visits may provide more accurate representations of their typical out of office blood pressures. Post-physician AOBP monitoring without an MA present may be particularly useful for African American patients and lead to greater blood pressure measurement accuracy, diagnosis, and improved hypertension treatment.

### Conflicts of Interest

The authors declare no conflicts of interest.
